# The Role of Stefin B in Neuro-inflammation

**DOI:** 10.3389/fncel.2015.00458

**Published:** 2015-12-08

**Authors:** Nataša Kopitar-Jerala

**Affiliations:** Department of Biochemistry, Molecular and Structural Biology, Jožef Stefan InstituteLjubljana, Slovenia

**Keywords:** cystatins, EPM1, inflammation, microglia, TLR, NLRP3 inflammasome, ROS

## Abstract

Stefin B (cystatin B) is an endogenous cysteine cathepsin inhibitor localized in the cytosol, mitochondria and nucleus. Its expression is upregulated upon macrophage activation and cellular stress. Mutations in the gene of stefin B are associated with the neurodegenerative disease known as Unverricht-Lundborg disease (EPM1). It was reported that early microglial activation precedes neuronal loss in the brain of the stefin B-deficient mice, implying a role of the inhibitor at the cross-talk between microglia and cerebellar cells. Detailed analysis of microglial activation in stefin B-deficient microglia showed a significantly higher proportion of both pro-inflammatory M1 and anti-inflammatory M2 microglia in stefin B-deficient mouse brain compared with control mice. In our recent work, we demonstrated that stefin B-deficient mice were significantly more sensitive to the lethal lipopolysaccharide (LPS)-induced sepsis, due to increased caspase-11 expression and secreted higher amounts of pro-inflammatory cytokines IL-1β and IL-18. Upon LPS stimulation, stefin B was targeted into the mitochondria, and the lack of stefin B resulted in the increased destabilization of the mitochondrial membrane potential and mitochondrial superoxide generation. The increased caspase-11 gene expression and better pro- inflammatory caspase-1 and -11 activation determined in stefin B deficient bone marrow-derived macrophages resulted in enhanced non-canonical inflammasome activation. Since signaling pathways in macrophages could be compared to the ones in microglia we propose that inflammasome activation could play an important role in the pathogenesis of EPM1.

## Introduction

Inflammation is a protective and tightly regulated immune response to tissue damage or pathogen invasion (Chovatiya and Medzhitov, 2014). In the central nervous system (CNS), this process is known as neuroinflammation and is characterized by the activation of the microglia and astrocytes population (Aguzzi et al., 2013). The innate immune response is triggered upon the recognition of pathogen-associated molecular patterns (PAMPs), derived from invading pathogens, and danger-associated molecular patterns (DAMPs), induced as a result of endogenous stress, by pattern-recognition receptors (PRRs; Akira et al., 2006). Activation of PRRs by PAMPs or DAMPs triggers signaling cascades that promote gene transcription by nuclear factor-κB (NF-κB), activator protein 1 (AP1), and interferon regulatory factors (IRFs) and results in the production of pro-inflammatory cytokines, interferons, and other pro-inflammatory proteins (Akira et al., 2006; Kawai and Akira, 2009). DAMPs correspond to endogenous ligands that are released by dying or damaged cells after cellular stress and can be recognized by PRRs such as membrane-bound toll-like receptors (TLRs) or cytosolic nucleotide-binding domain and leucine-rich repeat-containing (NLR), the RIG-I-like receptor (RLR), the AIM2-like receptor (ALR; Medzhitov, 2007; Moresco et al., 2011; Franchi et al., 2012).

In the CNS, PRRs are primarily expressed by microglia, macrophages and astrocytes. These receptors are either membrane-bound and sense extracellular or endosomally located signals (TLRs) or are located within the cytoplasm and sense intracellular signals (NLRs). Recently, it was proposed that TLRs have an important role in the crosstalk between neurons and glial cells in the CNS. TLR signaling was linked to neurogenesis, it was also found to be involved in the pathogenesis of neurodegenerative diseases (Heneka et al., 2014). Only cytosolic receptors are involved in the formation of inflammasomes. The inflammasome is an intracellular multimolecular complex for the activation of inflammatory caspases-1 and -11 which leads into the cleavage and secretion of IL-1β and IL-18 and cell death called – pyroptosis (Martinon and Tschopp, 2007; Lamkanfi and Dixit, 2014). Caspases-1 and -11 both induce pyroptosis, but only caspase-1 processes IL-1β and IL-18 (Kayagaki et al., 2011). The nucleotide binding and oligomerization domain-like receptor family pyrin domain containing 3 (NLRP3) inflammasome, which is composed of NLRP3, the adaptor molecule apoptosis-associated speck-like protein containing a caspase recruitment domain (ASC) and the cysteine protease caspase-1, is one of the most studied inflammasomes with responses to various endogenous and exogenous danger signals (Latz et al., 2013). The priming step, that up-regulates NLPR3 pro-IL-1β gene expression, provides TLR signaling (Bauernfeind, 2009). Once primed, NLRP3 can respond to its stimuli and assemble the NLRP3 inflammasome. Stimuli that induce NLRP3 inflammasome assembly include ATP, pore-forming toxins, crystalline substances, nucleic acids, hyaluronan, and fungal, bacterial, or viral pathogens (Latz et al., 2013; Lamkanfi and Dixit, 2014). It has been proposed that phagocytosis of crystalline or particulate structures triggers lysosomal destabilization and subsequent release of the lysosomal cathepsins into the cytosol, and subsequently activates NLRP3 inflammasome (Halle et al., 2008). However, it is not known yet if the cathepsins interact directly with the inflammasome or the process involves molecules activated by the cathepsins. Recent studies have revealed a role for reactive oxygen species (ROS) of mitochondrial origin in the promotion of NLRP3 inflammasome activation (Nakahira et al., 2011; Zhou et al., 2011). Several reports showed that caspase-8 localizes and binds to ASC specks, indicating that caspase-8 is an important component of the inflammasome complex (Man, 2013; Sagulenko, 2013). In addition to the canonical [lipopolysaccharide (LPS) and ATP] NLRP3 inflammasome activation, a non-canonical inflammasome activation was described (Kayagaki et al., 2011; Rathinam et al., 2012; Broz and Monack, 2013). Canonical inflammasomes convert procaspase-1 into the catalytically active enzyme, whereas an undefined non-canonical inflammasome promotes activation of procaspase-11 (Lamkanfi and Dixit, 2014). The mouse caspase-11 (gene name *Casp4)* has 46% similarities to caspase-1 and is orthologous to human caspases-4 and -5 (Wang et al., 1996; Kajiwara, 2014). Non canonical inflammasomes could be activated by Gram-negative, but not by Gram-positive, bacteria, indicating that a specific factor from Gram-negative bacteria – LPS is required (Broz et al., 2012; Rathinam et al., 2012). In addition, caspase-11 detected intracellular LPS and some intracellular bacteria, directly mediate cell death and IL-1α secretion by a TLR4-independent mechanism (Hagar et al., 2013; Lamkanfi and Dixit, 2014). The non-canonical inflammasome pathway caspase-11 can interact with caspase-1 and forms a heterodimeric complex. It could induce a lytic cell death similarly to caspase-1; however, it cannot by itself trigger IL-1β/-18 processing (Wang et al., 1998; Kayagaki et al., 2011). Only caspase-11-deficient mice, but not caspase-1-deficient mice were protected from endotoxic shock (Wang et al., 1998; Kayagaki et al., 2011). The CNS is particularly sensitive to IL-1β and IL-18 signaling because multiple neural cell types in the CNS express receptors for these cytokines (Allan et al., 2005; Alboni et al., 2010).

The goal of the present review is to describe recent advances in neuroinflammation and the role of stefin B in the process.

## Cells of the Immune System in CNS

Microglia are CNS resident myeloid cells of embryonic hematopoietic origin and comprise approximately 12% of cells in the brain (Aguzzi et al., 2013). Other CNS resident cells descend from neuroepithelial stem cells and are categorized as neurons and macroglia, with glia further subdivided into astrocytes and oligodendrocytes.

Astrocytes maintain CNS homeostasis and provide neuronal support in healthy conditions; moreover, astrocytes can undergo diverse phenotypic changes that may be protective or causative with regard to pathology (Sofroniew and Vinters, 2010). Astrocytes can produce numerous inflammatory molecules like cytokines, chemokines, growth factors, and nitric oxide (NO). Analysis of astrocyte transcriptome profiles indicates that astrocyte exposure either *in vivo* or *in vitro* to PAMPs such as LPS turns astrocyte transcriptome changes toward pro-inflammatory and potentially cytotoxic profiles (Hamby, 2012; Zamanian, 2012). Although astrocytes may undergo diverse phenotypic changes and secrete pro-inflammatory molecules, a recent study reported that NLRP3 inflammasome was expressed and functional only in mouse brain microglia, but not in astrocytes (Gustin et al., 2015). However, microglial–astrocyte interactions are important in the CNS innate immunity.

Microglia is a unique myeloid cell population, derived from primitive myeloid progenitors that arise before embryonic day 8, before vascularization or definitive hematopoiesis in the embryo (Ginhoux et al., 2010). Its density varies by brain region, they are localized mostly in the grey matter, with the highest concentrations being found in the hippocampus, olfactory telencephalon, basal ganglia, and substantia nigra (Lawson et al., 1990). Upon localization, microglia acquires a compact or ramified phenotype (Lawson et al., 1990; Block et al., 2007). In their resting state microglia have ramified morphology, and monitor the brain environment. In response to immunological stimuli or brain injury the cells are activated (Saijo and Glass, 2011). Activated microglia acquire a compact phenotype and up-regulate several surface molecules like major histocompatibility complex (MHC) molecules, chemokine receptors and several other markers (Rock, 2004). Under other circumstances, however, microglia become over-activated and can induce significant and highly detrimental neurotoxic effects by the excess production of a large array of cytotoxic factors such as superoxide (Colton and Gilbert, 1987), NO (Moss and Bates, 2001), and tumor necrosis factor-α (TNFα; Lee et al., 1993). In some cases, microglial responses could also be protective to the CNS (Lalancette-Hebert et al., 2007; Vinet et al., 2012). Gene expression and morphological changes associated with microglial activation have been extensively studied (Prinz and Priller, 2014). Several TLRs are expressed on the microglial membrane and signaling induced by TLR activation results in production of neurotoxicity and could contribute to the microglial response to neuronal damage. Activation of TLR2, TLR4, and TLR9 induces microglial production of NO through multiple ligands (Ebert, 2005). TLR9 recognizes single-stranded unmethylated CpG-DNA (bacterial DNA), which stimulates an increase in the production of microglial NO and TNFα (Olson and Miller, 2004; Ebert, 2005). TLR4 together with CD14 is implicated in brain inflammation and microglial activation in response to endotoxemia (Chakravarty and Herkenham, 2005). Monocyte-derived macrophages are classified as M1, M2a, M2b, and M2c subsets (Gordon and Taylor, 2005; Geissmann et al., 2010). It is possible that microglia also transcribe activation-dependent genes, like macrophages. Both microglia and macrophages share several similarities, they are both myeloid-derived cells; however, there are also some differences between the two cell types. Some common markers used for microglial identification such as CD11b, CD11c, and CX3CR1, could be found in microglial cells as well as in monocytes, macrophages, and dendritic cells. The difference in the expression level of cell membrane tyrosine phosphatase CD45 may be used to discriminate CD45^low^ microglia from CD45^high^ blood-derived cells by flow cytometry (Sedgwick et al., 1991; Ford et al., 1995). However, the signaling pathways in NLRP3 inflammasome activation are comparable between macrophages and microglia (Halle et al., 2008). Microglia clear apoptotic cells and are involved in both elimination and maintenance of synapses, they use their fine processes to monitor for dysfunctional synapses, which they are able to eliminate by phagocytosis (Wake et al., 2009). They also promote synaptic activity by secretion of brain-derived neurotrophic factor (BDNF), a molecule that is essential for learning-dependent synapse formation (Parkhurst, 2013). Moreover, microglia could modulate adult neurogenesis in the brain (Vukovic et al., 2012). Some studies suggested that microglial cells not only have a scavenger role during development but can also promote the death of some neuronal populations (Marin-Teva, 2004). Several studies have reported NLRP3 activation in microglia or CNS macrophages, although NLRP3 has also been proposed to function in neurons (Compan, 2012; Ramos, 2012). The activation mechanisms reported for NLRP3 activation in macrophages, such as ROS production, K^+^ eﬄux and endosomal rupture, also apply to NLRP3 activation within microglia (Halle et al., 2008; Hoegen, 2011; Heneka, 2013). Not only caspase-1, but also caspase-11 is expressed in microglial cells and could contribute to inflammasome activation (Lee et al., 2001; Kim et al., 2003).

## Cystatins in Inflammation

Cystatins were initially characterized as inhibitors of lysosomal cysteine cathepsins, however, in recent years some alternative functions for cystatins have been proposed. Cystatins possessing inhibitory function are members of three families, family I (stefins), family II (cystatins), and family III (kininogens; Kopitar-Jerala, 2006; Turk et al., 2008).

The cystatins (cysteine proteinase inhibitor) are reversible and tight-binding inhibitors of the papain (C1) and legumain (C13) families of cysteine proteases and have significant similarities in amino acid sequence (Barrett, 1981; Barrett et al., 1986). The inhibitory profile of a particular cystatin is rather specific, despite significant sequence homologies (Turk et al., 2008). Type 1 cystatins – stefins are mostly intracellular cystatins present in the cytosol and the nuclei (Abrahamson et al., 1986). They are single-chain polypeptides ∼100 amino acid residues long, are synthesized without signal peptides and do not possess any disulfide bonds or carbohydrate side-chains. Recently, we reported the mitochondrial localization of stefin B (Maher et al., 2014a). Type 2 cystatins are mainly extracellular, secreted proteins. They are synthesized with 20–26 residue long signal peptides and most of them are found in physiologically relevant concentrations in body fluids (Abrahamson et al., 1986; Kopitar-Jerala, 2006; Turk et al., 2008). They contain disulphide bridges and may be phosphorylated (Laber et al., 1989). Type II cystatins also possess a second reactive site for inhibition of the C13 family of cysteine proteases (legumain; Alvarez-Fernandez et al., 1999). Cystatin C was found upregulated in the serum of patients with autoimmune diseases like systemic lupus erythematosus (Lertnawapan et al., 2012). Moreover, cystatin F was found abundant in the cells of the immune system: macrophages and dendritic cells and the cells involved in target cell killing (NK cells and cytotoxic T cells (CTLs; Halfon, 1998; Ni et al., 1998; Obata-Onai et al., 2002). It was also found in the microglial cells and monocyte/macrophages in the CNS. Cystatin F is expressed as a di-sulfide-linked dimer (Cappello et al., 2004) and translocated to endolysosomes where it regulates cathepsin activity. Cystatin F transport to endolysosomes depends on its *N*-linked glycosylation and it was reported that the secreted dimeric cystatin F could be internalized and activated by the mannose-6-phosphate receptor system (Colbert et al., 2009). After proteolytic removal of its N-terminal part, cystatin F becomes a potent inhibitor of cathepsin C with the potential to regulate pro-granzyme processing and cell cytotoxicity (Hamilton et al., 2008). Recently, we demonstrated that cathepsin V in IL-2 stimulated NK cells could process cystatin F (Maher et al., 2014b). In cytotoxic cells, cystatin F, therefore, appears as a key regulator of granzyme processing and consequently cell cytotoxicity.

Type 3 cystatins are high molecular weight (60–120 kDa) multidomain proteins and have three tandemly repeated type 2-like cystatin domains (Salvesen et al., 1986). The mammalian cystatins belonging to this type are the kininogens (Ohkubo et al., 1984). Cystatins in immune cells have been reported to participate in the release of nitric oxide, phagocytosis, and expression of cytokines (Kopitar-Jerala, 2006; Magister and Kos, 2013; Maher et al., 2014a).

## Stefin B and EPM1 1

Stefin B belongs to the type one cystatins and is located in the cytosol, mitochondria, and nucleus where it protects cells from the detrimental release of the lysosomal cysteine cathepsins. In the nucleus, stefin B interacts with nucleosomes, specifically with histones H2A.Z, H2B, and H3 and cathepsin L (Ceru et al., 2010). Goulet et al. (2004) has shown that only shorter procathepsin L isoforms translocate to the nucleus and stimulate processing of the CUX1 transcription factor at the G_1_/S transition of the cell cycle. Stefin B-deficient mouse embryonic fibroblasts entered S phase earlier than wild type mouse embryonic fibroblasts. In contrast, increased expression of stefin B in the nucleus delayed cell cycle progression in T98G cells. The delay in cell cycle progression was associated with the inhibition of cathepsin L in the nucleus, as judged from the decreased cleavage of the CUX1 transcription factor (Ceru et al., 2010). Moreover, we have shown that stefin B overexpression in the nucleus delayed not only cell cycle progression, but also caspase activation (Sun et al., 2012). Mutations in the gene encoding stefin B (either through a multiplied repeat unit in the promoter or through point mutations in the structural gene) are present in both alleles of the gene in patients with a form of progressive myoclonus epilepsy of Unverricht-Lundborg type (EPM1; Pennacchio et al., 1996; Lalioti et al., 1997; Pennacchio et al., 1998). EPM1 is an autosomal recessively inherited neurodegenerative disease, characterized by the cerebellar granule neurons apoptosis, progressive ataxia and myoclonic epilepsy (Joensuu et al., 2008). In lymphoid cells of EPM1 patients, increased cathepsin activity, due to reduced expression of stefin B was reported (Rinne et al., 2002), we determined increased overall cathepsin activity in untreated, as well as in classically activated stefin B-deficient bone marrow-derived macrophages (BMDMs) compared to WT cells (Maher et al., 2014c).

## Mouse Model of EPM1

Stefin B-deficient mice develop myoclonic seizures by one month of age and progressive ataxia by six months of age (Pennacchio et al., 1998). Houseweart et al. (2003) reported that the removal of cathepsin B from stefin B-deficient mice greatly reduced the neuronal apoptosis, but did not rescue the ataxia and seizure phenotype. Moreover, stefin B deficiency was implicated in the impaired redox homeostasis, resulting in a pronounced oxidative stress-induced cell death and neurodegeneration (Lehtinen et al., 2009). Thymocytes from stefin B deficient mice were significantly more sensitive to apoptosis induced with the inhibitor of protein kinase C, staurosporin (Kopitar-Jerala et al., 2005). Manninen et al. examined in detail the spatiotemporal dynamics of the brain atrophy in stefin B-deficient mice (Manninen et al., 2014). They showed progressive but non-uniform volume loss of the stefin B-deficient mouse brains, indicating that different neuronal populations possess distinct sensitivity to the damage, as a consequence of stefin B deficiency. The authors suggested that the white matter damage in the brain of stefin B-deficient mice was secondary to glial activation and neurodegeneration (Manninen et al., 2014). Another report showed that the early microglial activation precedes neuronal loss in the brain of the stefin B deficient mice, implying a role of the inhibitor at the cross-talk between microglia and cerebellar cells (Tegelberg et al., 2012).

Joensuu et al. (2014) analyzed the gene expression changes in the cerebellum of pre-symptomatic and symptomatic stefin B -deficient mice and in cultured stefin B-deficient cerebellar granule cells. Already in the cerebellum of pre-symptomatic stefin B-deficient mice (7 days after the birth), multiple changes in gene expression related to synapse maturation, development, and function during postnatal maturation were observed. More prominent changes were reported in the GABAergic signaling pathway (Joensuu et al., 2014). GABA plays a central role in controlling neuronal development and connectivity and defective GABAergic signaling in the cerebellum of stefin B deficient mice underlines a mechanism for ataxia in these mice (Grusser-Cornehls and Baurle, 2001). At a later stage (30 days after the birth), in symptomatic stefin B-deficient mice, the authors reported the upregulation of immune response genes, in line with the results showing early glial activation that preceded neuronal degeneration (Tegelberg et al., 2012). Moreover, Joensuu et al. (2014), reported the upregulation of the genes involved in cell cycle progression, in stefin B-deficient granule neurons. We have shown that the interactions of stefin B with cathepsin L in the nucleus influence cell cycle progression into the S phase (Ceru et al., 2010). We cannot exclude the possibility that the impaired cathepsin regulation in the synapses could lead to morphological and functional changes observed in stefin B-deficient mice (Joensuu et al., 2014). For example, cathepsin B-like immunoreactivity was observed at synaptic sites and myristoylated-alanine-rich C-kinase substrate (MARCKS), a known substrate of cathepsin B, was specifically degraded in response to intense NMDA receptor stimulation (Graber et al., 2004). Previously, we reported the increased cleavage of MARCKS in the brains and macrophages of stefin B-deficient mice, when compared to cells and tissue from control wild-type animals (Kopitar-Jerala and Turk, 2007).

## Stefin B, EPM1, and Innate Immune Response

The increased expression of inflammatory genes indicates that neuro-inflammation, together with neuronal dysfunction, plays a crucial role in pathology of EPM1. Pro-inflammatory chemokines and cytokines, highly expressed in symptomatic stefin B-deficient mice were reported to lower the seizure threshold and may thus contribute to recurrent excitation in epilepsy (Devinsky et al., 2013). Okuneva et al. (2015) reported significantly higher stefin B mRNA expression in microglia than in neurons or astrocytes, which is in line with our observation that stefin B is highly upregulated in activated macrophages (Maher et al., 2014c). In pre-symptomatic stefin B-deficient mice compared to control animals the ratio of M1/M2 microglia is skewed towards M2 type, but towards M1 type in symptomatic mice. In addition, a heightened expression of both pro-inflammatory inducible nitric oxide synthase (iNOS), anti-inflammatory arginase 1 (ARG1) and chemokine release was detected (Okuneva et al., 2015). Interestingly, MHCII surface expression was suppressed. We have reported that IFN-γ and LPS-activated stefin B-deficient BMDMs produced higher amounts of NO, and expressed more iNOS than WT BMDMs. IL-10 is a potent anti-inflammatory cytokine that is crucial for dampening the inflammatory response after pathogen invasion and acts to protect the host from excessive inflammation (Fiorentino et al., 1991). We showed decreased expression of IL-10 in BMDMs of stefin B deficient mice, due to impaired STAT3 signaling (Maher et al., 2014c). IL-10 plays an essential role in mediating inflammatory processes not only in the cells of immune system, but also in the brain (Zocchia et al., 1997). It has been demonstrated that it increases the survival of cerebellar granule cells by blocking caspase-3-like activity (Bachis et al., 2001). It is tempting to speculate that the decreased IL-10 expression in stefin B-deficient mice could contribute to the increased apoptosis in the cerebellum in EPM1.

**FIGURE 1:**
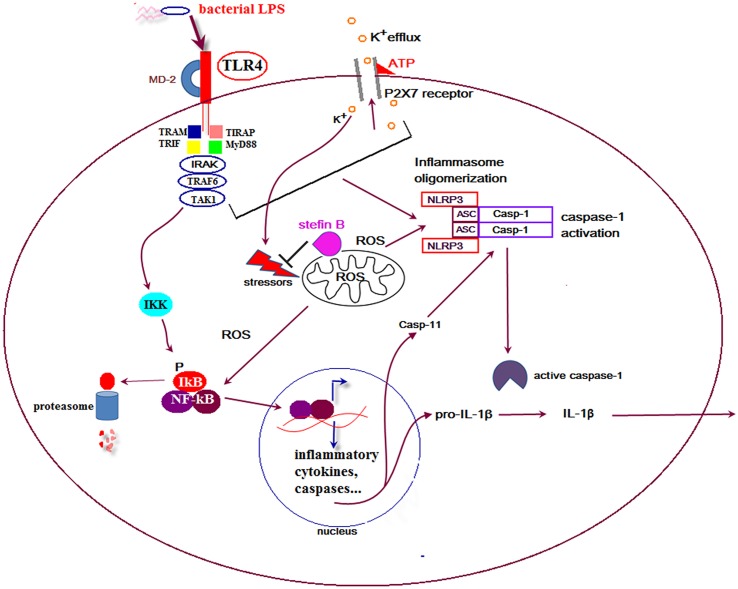
**Proposed model for the role of stefin B in non canonical inflammasome activation**. Upon lipopolysaccharide (LPS) stimulation stefin B is translocated from cytosol into mitochondria and protects mitochondrial membrane integrity. Stefin B deficiency resulted in the breakdown of mitochondria membrane potential and increased mtROS generation. The consequence of the increased mtROS detected in stefin B-deficient bone marrow-derived macrophages (BMDMs) upon LPS stimulation, was the increased nuclear factor-κB (NF-κB) activation and caspase-11 expression. Increased caspase-11 expression resulted in increased inflammasome activation and pro-inflammatory IL-1β and IL-18 secretion.

In the developing mouse cerebellum, Purkinje cells die and a majority of these neurons are engulfed by microglial cells. Interestingly, apoptosis of Purkinje cells in the cerebellum was strongly reduced by selective elimination of microglia and superoxide produced by microglia cells (Marin-Teva, 2004). In our recent work we showed that stefin B-deficient mice were significantly more sensitive to the lethal LPS-induced endotoxemia due to increased caspase-11 expression. The increased caspase-11 gene expression and better caspase-1 and -11 processing determined in stefin B-deficient BMDMs resulted in enhanced IL-1β and IL-18 processing and secretion (**Figure 1**). The increased cathepsin activity determined in stefin B deficient BMDMs was not essential for inflammasome activation, since treatment of BMDMs with the cathepsin inhibitor E-64d did not influence caspase-1 activation and IL-1β secretion. Upon LPS stimulation, stefin B was targeted to mitochondria, and the lack of stefin B resulted in the increased destabilization of mitochondrial membrane potential and mitochondrial ROS generation (Maher et al., 2014a). The induction of ROS in microglia may therefore play an important role in non-canonical inflammasome activation and cell death in the cerebellum in disease.

## Conclusion

This review summarizes recent discoveries that may contribute to the understanding of the role of stefin B in neuro-inflammation. Several studies, each from a different angle, have contributed a piece of the puzzle, a process we are trying to understand. Stefin B-deficient mice have proven to be a valuable tool to explore the function of the protein in the pathology of disease. During the past couple of years, several new data from microarray experiments, histology, as well as magnetic resonance imaging have revealed that neuro-inflammation is an essential process in the pathology of EPM1. Biochemical experiments in macrophages have contributed some hints regarding the signaling pathways in inflammasome activation. Signaling pathways in macrophages were compared to the ones in microglia and the expression of caspase-11 was strongly induced by activation of rat glial cells, as well as in astrocytes, with interferon-γ and LPS (Hur et al., 2001). The expression of caspase-11 in microglia may play an important role in non-canonical inflammasome activation and cell death in the cerebellum in disease. However, some questions still remain and some more pieces need to be added to complete the whole picture. Additional experiments will reveal if the inflammasome activation and caspase-11 expression are part of the pathology of EPM1.

## Conflict of Interest Statement

The author declares that the research was conducted in the absence of any commercial or financial relationships that could be construed as a potential conflict of interest.
